# Inhibition of nucleoporin member Nup214 expression by miR-133b perturbs mitotic timing and leads to cell death

**DOI:** 10.1186/s12943-015-0299-z

**Published:** 2015-02-15

**Authors:** Sumana Bhattacharjya, Kumar Singha Roy, Abira Ganguly, Shreya Sarkar, Chinmay K Panda, Dibyendu Bhattacharyya, Nitai P Bhattacharyya, Susanta Roychoudhury

**Affiliations:** Cancer Biology and Inflammatory Disorder Division, Indian Institute of Chemical Biology, Council of Scientific and Industrial Research, 4, Raja S.C. Mullick Road, Kolkata, 700 032 India; Tata Memorial Centre, ACTREC Sector 22, Navi Mumbai, Kharghar 410210 India; Department of Oncogene Regulation, Chittaranjan National Cancer Institute, 37, S.P. Mukherjee Road, Kolkata, 700 026 India; Crystallography and Molecular Biology Division, Saha Institute of Nuclear Physics, 1/AF, Bidhannagar, Kolkata, 700 064 India; Present address: Biomedical Genomics Centre, PG Polyclinic Building (3rd floor), 5, Suburban Hospital Road, Kolkata, 700 020 India

**Keywords:** Nucleoporin, Nup214, miR-133b, Mitosis, Apoptosis, Cell cycle, MicroRNA, Cancer, Head and neck cancer, Chromosomal abnormality

## Abstract

**Background:**

Nucleoporins mediate nucleocytoplasmic exchange of macromolecules and several have been assigned active mitotic functions. Nucleoporins can participate in various mitotic functions like spindle assembly, kinetochore organisation and chromosome segregation- important for genome integrity. Pathways to genome integrity are frequently deregulated in cancer and many are regulated in part by microRNAs. Indeed, altered levels of numerous microRNAs have frequently been associated with tumorigenesis. Here, we unveil a microRNA-mediated regulation of the nucleoporin Nup214 and its downstream effect on genome integrity.

**Methods:**

Databases/bioinformatic tools such as miRBase, Oncomine and RNAhybrid predicted Nup214 as a miR-133b target. To validate this, we used luciferase reporter assays, Real-Time PCR and immuno-blotting. Flow cytometry and immuno-blots of mitotic markers were used to analyse cell cycle pattern upon thymidine synchronization and miR-133b treatment. Mitotic indices and chromosomal abnormalities were assessed by immuno-fluorescence for FITC-tagged phospho-H3 as well as video-microscopy for GFP-tagged histone H4. Annexin V/propidium iodide staining, caspase3/PARP cleavage and colony formation assays were done to investigate cell death upon either miR-133b transfection or NUP214 knockdown by siRNA. UPCI:SCC084, HCT116, HeLa-H4-pEGFP and HEK293 (human oral squamous cell carcinoma, colorectal, cervical carcinomas and embryonic kidney cell lines, respectively) were used. miR-133b and NUP214 expressions were validated in cancer cell lines and tissues by Real-Time PCR.

**Results:**

Examination of head and neck tumour tissues and cancer cell lines revealed that Nup214 and miR-133b expressions are negatively correlated. *In vitro*, Nup214 was significantly downregulated by ectopic miR-133b. This downregulation elevated mitotic indices and delayed degradation of mitotic marker proteins cyclinB1 and cyclinA and dephosphorylation of H3. Moreover, this mitotic delay enhanced chromosomal abnormalities and apoptosis.

**Conclusions:**

We have identified NUP214, a member of the massive nuclear pore complex, as a novel miR-133b target. Thus, we have shown a hitherto unknown microRNA regulation of mitosis mediated by a member of the nucleoporin family. Based on observations, we also raise some hypotheses regarding transport-dependent/independent functions of Nup214 in this study. Our results hence attempt to explain why miR-133b is generally downregulated in tumours and lay out the potential for Nup214 as a therapeutic target in the treatment of cancer.

**Electronic supplementary material:**

The online version of this article (doi:10.1186/s12943-015-0299-z) contains supplementary material, which is available to authorized users.

## Background

MicroRNAs (miRNA) are endogenous, non-coding RNAs that direct gene repression by inhibiting the mRNA stability or translation [1]. Evolutionarily conserved, they frequently target 3′ untranslated regions (UTRs) of target transcripts [2]. A variety of cellular events such as proliferation, differentiation and apoptosis are controlled by miRNAs- thus implicating them in tumorigenesis [3,4]. Moreover, altered expression levels of miRNAs have been associated with myriad kinds of cancers [4]. However, elucidation of molecular pathogenic pathways involving these miRNAs has not kept pace with the fast-increasing number of association studies between altered miRNA expressions and different types of cancers.

Meanwhile, a vast, complex network of signalling pathways mediate tumour progression and all such signals have to enter the nucleus through the only gateway- the nuclear pore complex (NPC). The NPCs regulate the nucleocytoplasmic trafficking of macromolecules and are massive protein complexes within the cell, composed of multiple copies of about 30 different nucleoporins (Nups) [5,6]. Cell division demands exquisite control over the arrangement and location of events and failure to observe this control has dire consequences, including chromosome mis-segregation and aneuploidy [7]. The activation or inactivation of a series of mitotic regulators determines the timing, but the completion of one event [for example, nuclear envelope breakdown (NEBD)] might also provide a signal for the beginning of the next, maintaining a chronological sequence through mitosis. Unveiling of surprising links between the nuclear transport machinery, kinetochore and the spindle assembly checkpoint (SAC) besides canonical mitotic proteins [8] support this idea [9,10]. Certain proteins that localize to the NPC in interphase can relocalize to the kinetochores during mitosis, wherein they are required for the alignment of chromosomes to the metaphase plate. The sequestration of kinetochore proteins at the NPC might therefore ensure that the kinetochores cannot function until NEBD has been completed [11]. For instance, the checkpoint proteins Mad1, Mad2 and Mps1 are all associated with the NPCs in vertebrate cells [12]. Remarkably, this ‘dual citizenship’ is reciprocal: Nups also localize to both nuclear pores and kinetochores, and seem to perform distinct functions in each location [11]. Hence, the mayhem that can occur from the disruption of even a single Nup function is fathomable. Moreover, the dynamics of NPCs are tightly regulated during the cell cycle [13]. Although it was assumed that Nups were just structural components of the NPC, this view was transformed by the surprising discovery that Nup107 and Nup133 localize to the spindle poles and to the kinetochores during mitosis [14]. In this context, several Nups such as Nup153, Nup 107–160 complex, Tpr, Rae1 and Nup88 have been implicated in mitotic processes such as chromosome condensation, sister-chromatid cohesion, kinetochore assembly and spindle formation. Naturally, altered cellular levels of Nups can thus contribute to chromosomal instability (CIN), aneuploidy and tumorigenesis [15]. In this regard, it is well-documented that CIN and aneuploidy are hallmarks of many different malignancies [16,17]. Furthermore, deregulation of the cell cycle in general and mitosis in particular is known to be frequently causal to CIN [18,19].

Our earlier data predicted a large number of mitotic targets of miRNAs deregulated in head and neck squamous cell carcinoma (HNSCC) [20]. Of these, NUP214 was observed to be a potential target of hsa-miR-133b, a downregulated miRNA in HNSCC. The human proto-oncogene NUP214 is reported to have a dual role in nuclear protein import and mRNA export. The fact that mice embryos homozygous for the disrupted NUP214 allele die *in utero* mirrors the essentiality of Nup214 for cell survival [21]. Moreover, Nup214 localizes to both the cytoplasmic and nucleoplasmic sides of the NPC in over-expressing cells [22]. Additionally, it is also reported to be recruited to the spindles during mitosis [23,24]. However, the exact function of Nup214 in mitosis remains elusive till date. Moreover, to the best of our knowledge, the role of miRNAs in the regulation of Nups has not been elucidated yet. Given the fact that both Nups and miRNAs are crucial to genome integrity, here we aim to elucidate how the regulation of one (Nup) by the other (miRNA) modulates this very genome integrity as well as its possible implications in tumorigenesis.

In the current study, we present NUP214 as a novel target of miR-133b. We also show that downregulation of Nup214 by miR-133b perturbs the normal mitotic progression. This perturbation subsequently gives rise to chromosomal abnormalities and leads to cell death by apoptosis. Through our study, we also raise some interesting questions about the possible locations and functions of Nup214 during mitosis.

## Results

### In silico analysis predicts miR-133b binding site on NUP214 3′UTR

Through an in silico prediction for a number of miRNAs by miRBase, we had shown that NUP214 is a putative target of miR-133b [20]. Briefly, expression status of potential miR-133b target genes were determined by Oncomine database analysis and those which were reported to be upregulated in HNSCC (inverse to miR-133b underexpression) were selected. NUP214 was found to feature in this list of targets. Indeed, RNAhybrid analysis revealed that the 3′UTR of NUP214 has a miR-133b recognition site at position 9 to 36 (Additional file 1A and B). It was also found that the NUP214 expression levels are elevated in a number of cancers (Additional files 1C and 2).

### miR-133b and Nup214 expression levels are negatively correlated in cancer cell lines and primary HNSCC tissue samples

**Figure 1:**
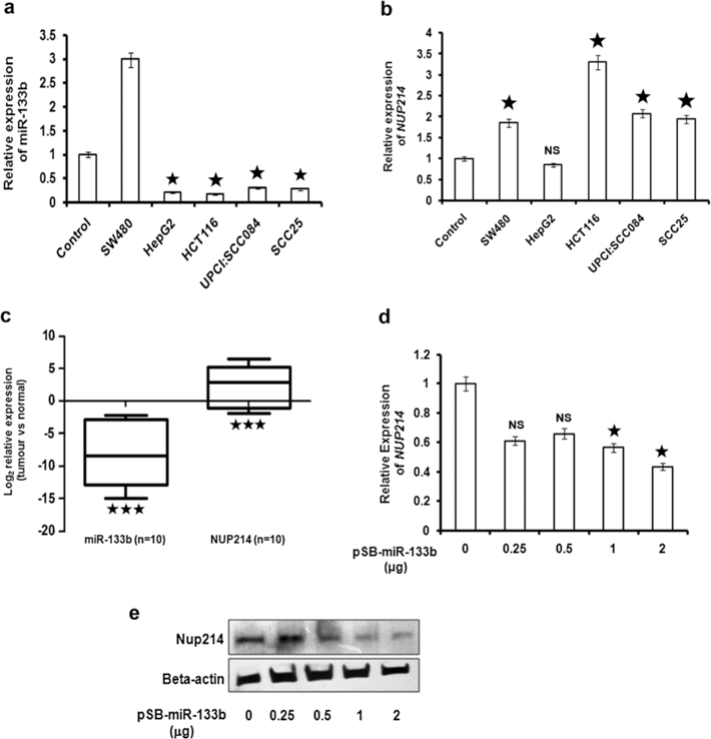
**miR-133b targets NUP214 and their expressions are inversely related. (a)** Expression of endogenous miR-133b in different cancer cell lines compared to control oral epithelial cells. **(b)** Corresponding levels of endogenous NUP214 in the same cell lines compared to control oral epithelial cells. **(c)** Reciprocal relation of miR-133b and NUP214 expressions in HNSCC tissues. Relative expression values were calculated and the data plotted as shown. ‘n’ represents number of tumour samples; asterisk denotes significant difference of expression in tumour versus normal samples with P-value < 0.0005. **(d)** NUP214 is reduced at the mRNA level in presence of ectopic miR-133b. UPCI:SCC084 cells were transiently transfected with 0, 0.25, 0.5, 1 and 2 μg of pSB-miR-133b. **(e)** Representative picture showing Nup214 protein level decreases upon ectopic miR-133b expression. UPCI:SCC084 cells were transiently transfected with 0, 0.25, 0.5, 1 and 2 μg of pSB-miR-133b. Cell lysates were prepared followed by Western blot with antibodies against Nup214 and β-actin. For **(a)** and **(c)**, total RNA from oral swab (control), UPCI:SCC084, SCC25, SW480, HCT116 cells and HNSCC tumour and normal tissues were reverse transcribed using miR-133b-specific stem-loop primers. cDNAs were subjected to RT-PCR. Relative expression values were normalized to those of miR-17-5p or (for cell lines) or U6 snRNA (for tissues). For **(b)**, **(c)**, and **(d)**, cDNA was subjected to RT-PCR using NUP214-specific primers. Relative expression values were normalized to those of GAPDH (for cell lines) or 18 s rRNA (for tissues). For **(a)**, **(b)** and **(d)**, data represent three independent experiments and are shown as average ± standard deviation (S.D). For **(e)** images are representative of three independent experiments. For all figures, asterisk denotes significant change when compared to either control or miR-133b-treated condition with p value <0.05; NS – not significant.

miR-133b has been reported to be downregulated in several SCC cell lines [25]. Preliminarily, we began with testing and reconfirming that miR-133b expression is indeed low in two SCC cell lines UPCI:SCC084 and SCC25 than in normal oral epithelial cells (Figure 1a). Conversely, we found that the NUP214 transcript levels are also higher in the same cell lines (Figure 1b). Moreover, this inverse expression pattern was observed in other cancer cell lines like HCT116 as well, with the exception of only SW480 (Figure 1a and b). We thus selected the UPCI:SCC084 and HCT116 cell lines to study the possible role of miR-133b in NUP214 regulation.

Similarly, the pooled data from ten HNSCC tissue samples demonstrated that indeed, miR-133b and Nup214 expressions are inversely related in these samples (Figure 1c). The relative expression table reveals a median ~300 fold decrease in miR-133b and ~7 fold increase in Nup214 expressions (Additional file 3). Together, this finding serves to support to an extent, our *in vitro* observations. Pathological details of these samples can be found in Additional file 4.

### miR-133b binds to the NUP214 3′UTR and negatively regulates its expression

In order to establish NUP214 as a *bona fide* target of miR-133b, we transfected UPCI:SCC084 cells with different doses of miR-133b expression-plasmid and measured NUP214 both at the mRNA and protein levels. We observed that the NUP214 transcript levels decreased in consonance with the increasing doses of the miRNA indicating that miR-133b regulates NUP214 at the post-transcriptional level (Figure 1d and Additional file 5A). Similarly, Nup214 also declined at the protein level upon ectopic miR-133b expression (Figure 1e and Additional file 5B). Moreover, the observation that the levels of another unrelated Nup, Nup98 that does not carry a recognition site for miR-133b, remained unaffected by miR-133b confirmed the specificity of this interaction (Additional file 5C). Additionally, the mitotic gene BUB1 (does not have a recognition-site for miR-133b) also remained unchanged upon miR-133b transfection which further strengthened this specificity (Additional file 5D-F). On the other hand, the NUP214 levels also remained unaltered in presence of another unrelated miRNA, miR-125b, which does not have a binding site on NUP214 (Additional file 5G-I).

**Figure 2:**
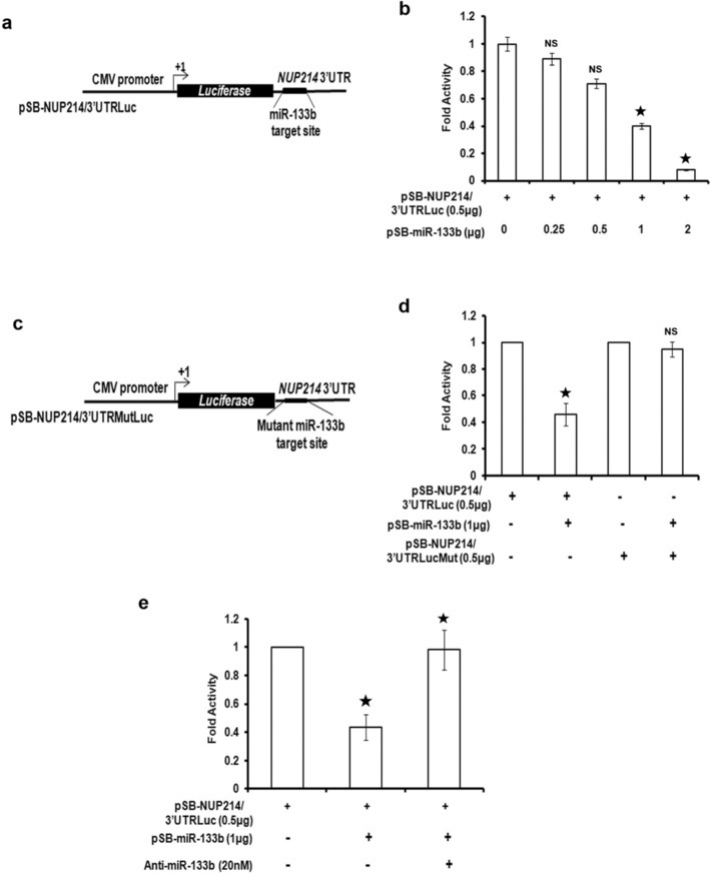
**Binding of miR-133b to NUP214 is specific. (a)** and **(c)** Cartoons showing the two NUP214 3′UTR constructs used for luciferase assay. **(a)** represents wild type NUP214 3′UTR downstream of luciferase gene while **(c)** shows NUP214 3′UTR where the target site for miR-133b is mutated. **(b)** miR-133b decreases luciferase activity in presence of NUP214 3′UTR. UPCI:SCC084 cells were transiently co-transfected with 0.5 μg pSB-NUP214/3′UTRLuc and 0, 0.25, 0.5, 1 or 2 μg of pSB-miR-133b. Protein lysates were prepared for luciferase assay. **(d)** Mutant NUP214 3′UTR prevents inhibition by miR-133b. UPCI:SCC084 cells were co-transfected with either 0.5 μg pSB-NUP214/3′UTRLuc, or pSB-NUP214/3′UTRMutLuc along-with 0 or 1 μg pSB-miR-133b. Protein lysates were prepared for luciferase assay. **(e)** Blocking miR-133b prevents NUP214 repression. UPCI:SCC084 cells were transiently transfected with only pSB-NUP214/3′UTRLuc (0.5 μg), or with pSB-NUP214/3′UTRLuc (0.5 μg) and pSB-miR-133b (1 μg), or with pSB-NUP214/3′UTRLuc (0.5 μg), pSB-miR-133b (1 μg) and anti-miR-133b (20 nM). Protein lysates were prepared for luciferase assay. For **(b)**, **(d)** and **(e)**, data represent three independent experiments and are shown as average ± S.D.

Next, we co-transfected UPCI:SCC084 cells with either pSB-NUP214/3′UTRLuc (Figure 2a) or pSB-NUP214/3′UTRMutLuc (Figure 2c) with increasing doses of miR-133b and measured the relative luciferase activity. In accord, a dose-dependent decrease in luciferase activity was observed for the 3′UTR containing the wild type miR-133b recognition site (Figure 2b). Contrastingly, no significant change in luciferase activity was observed for the mutant NUP214 3′UTR (Figure 2d). To further fortify these findings, we also co-transfected UPCI:SCC084 cells with an anti-sense inhibitor against miR-133b along with the miRNA and pSB-NUP214/3′UTRLuc. As expected, miR-133b was not available to bind to its target site on the NUP214 3′UTR and for this reason, was unable to restrain the luciferase activity (Figure 2e). Overall, these results prove that miR-133b binds to the NUP214 3′UTR and specifically downregulates its expression.

### Nup214 repression by ectopic miR-133b retards mitotic progression

**Figure 3:**
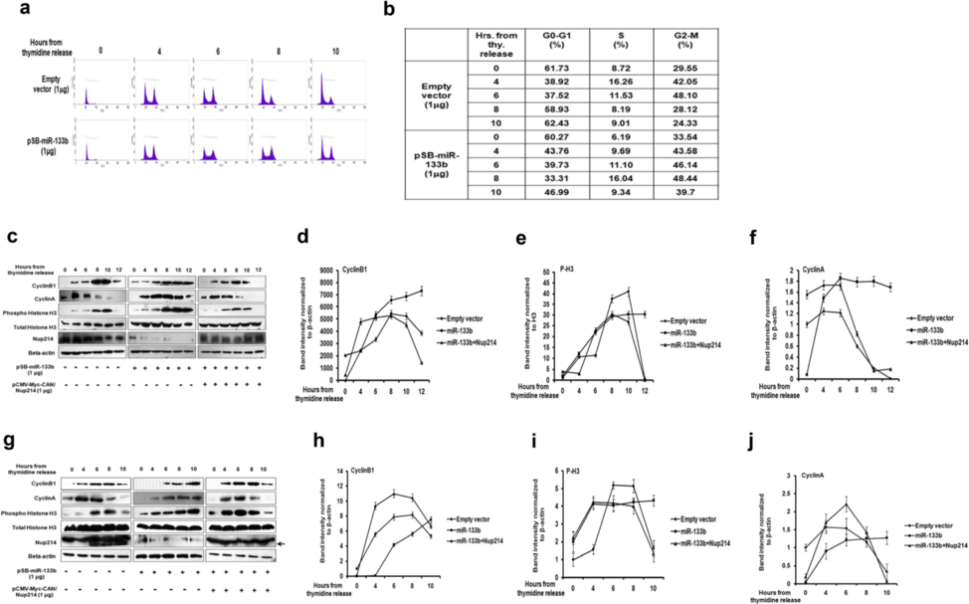
**miR-133b perturbs mitotic timing by downregulating Nup214. (a)** and **(b)** miR-133b halts cells at G2/M phase. UPCI:SCC084 cells were transiently transfected with 1 μg pSB-miR-133b or empty vector, synchronized and harvested at 0, 4, 6, 8 and 10 h from second thymidine release. DNA content was analysed by flow cytometry. **(a)** shows representative images while **(b)** shows the percentage of cells in each phase at each time-point. **(c)** and **(g)** Representative pictures showing miR-133b delays cyclinB1, cyclinA degradation and H3 dephosphorylation while Nup214 rescues the normal pattern even in presence of miR-133b. UPCI:SCC084 **(c)** and HCT116 **(g)** cells were transiently transfected with empty vector (1 μg), pSB-miR-133b (1 μg), or pSB-miR-133b (1 μg) and pCMV-Myc-CAN/Nup214 (1 μg), and synchronized. Cell lysates were prepared at 0, 4, 6, 8, 10 and 12 h from second thymidine release followed by Western blot with antibodies against cyclinB1, cyclinA, p-H3, Nup214 and β-actin. **(d)**, **(e)**, **(f)**, **(h)**, **(i)** and **(j)** Densitometric values of bands by ImageJ are plotted: cyclinB1 **(d, h)** and cyclinA **(f, j)** were normalized to β-actin. P-H3 **(e, i)** was normalized to total H3. All three proteins were further normalised to the respective protein expressions at 0 h in empty vector-treated cells. For **(a)**, **(c)** and **(g)**, images are representative of three different experiments while for **(d)**, **(e)**, **(f)**, **(h)**, **(i)** and **(j)**, data represent three independent experiments and shown as average ± S.D. Black arrow in **(g)** represents a non-specific band.

A number of Nups are known to be involved in different mitotic processes [15]. In view of the fact that we were interested in identifying miRNA-mediated regulatory pathways in mitosis, and also because the regulation of mitotic proteins at the import/export level is crucial, we initially investigated whether Nup214 repression by miR-133b could affect the cell cycle/mitotic progression. Toward this, we used three different strategies to examine the effect of ectopic miR-133b on the cell cycle progression of UPCI:SCC084 cells synchronized by thymidine treatment and released from G1 block. First, FACS analysis of vector-transfected cells revealed that their peak accumulation at the G2/M phase (48.10%) occurred at 6 h post-release. By 10 h from release, percentage of cells in G2/M reduced to 24.33% (Figure 3a and b, upper panels). In comparison, 48.44% of cells over-producing miR-133b were in G2/M at 8 h from release, and remained insignificantly changed (39.7%) even at 10 h post-release (Figure 3a and b, lower panels).

Secondly, we monitored the mitotic progression by examining the cyclic levels of cyclinB1 and phospho histone H3 (p-H3)- marker proteins for mitosis, in both UPCI:SCC084 and HCT116 cells. For vector-transfected UPCI:SCC084 cells, cyclinB1 peaked at 8–10 h post-release, after which it declined, manifesting a mitotic exit (Figure 3c, left panel and d). In contrast, upon ectopic miR-133b expression cyclinB1 did not degrade even at 12 h post-release implying that the cells were yet to exit from mitosis (Figure 3c, middle panel and d). Further, specificity of this mitotic regulation mediated by miR-133b was proved by the observation that rapid degradation of cyclinB1 could be reconstituted by ectopic Nup214 expression even in the presence of miR-133b (Figure 3c, right panel and d). Moreover, the observation made for cyclinB1 was replicated by the phosphorylation-dephosphorylation pattern of H3 (Figure 3c and f). Similarly, cyclinB1 and p-H3 were found to be stabilized by miR-133b and their degradation and dephosphorylation, respectively, were rescued by Nup214 in HCT116 cells as well (Figure 3g, h and i). These results together demonstrate that excess miR-133b arrests the cells in mitosis by suppressing the Nup214 levels. At this point, it is worth mentioning that owing to the absence of the 3′UTR recognition site for miR-133b in the Nup214 expression-plasmid, Nup214 is able restore the original phenotype even in presence of the miRNA.

**Figure 4:**
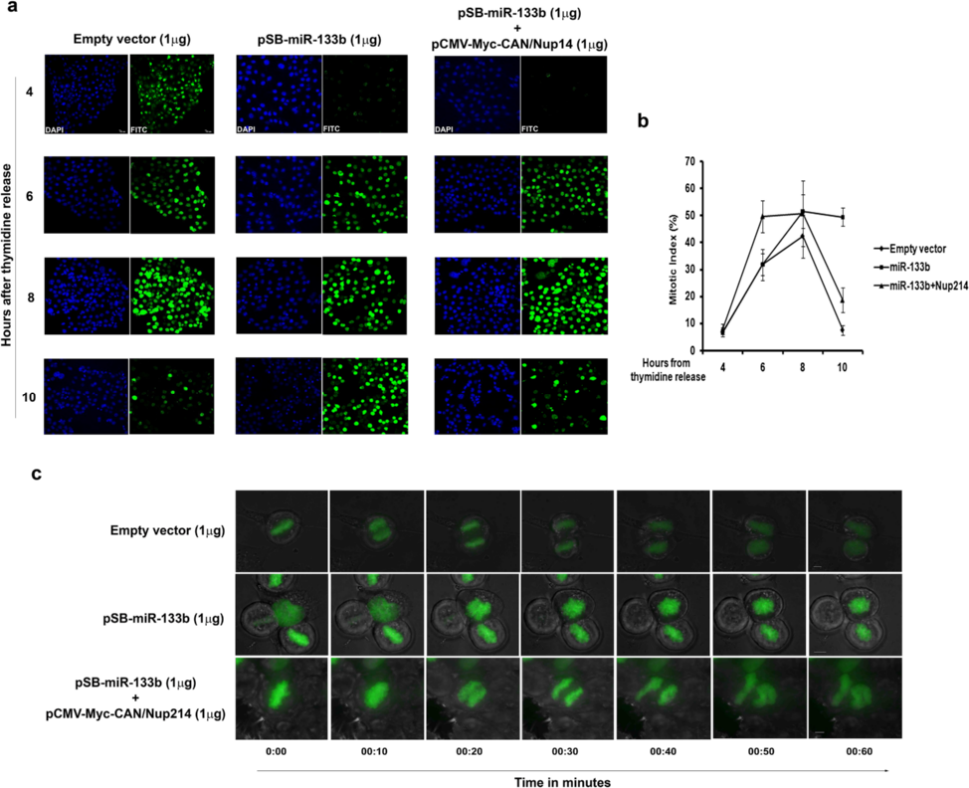
**miR-133b modulates mitotic timing. (a)** and **(b)** HCT116 cells were transiently transfected with empty vector (1 μg), pSB-miR-133b (1 μg), or pSB-miR-133b (1 μg) and pCMV-Myc-CAN/Nup214 (1 μg) and synchronized. At 4, 6, 8 and 10 h from second thymidine release, cells were fixed, stained with DAPI and visualized under a fluorescence microscope. Representative images are shown **(a)**. Only brightly-stained (FITC) cells were considered for MI evaluation. Scale bar represents 10 μm. Images represent 40× magnification. MI were calculated and plotted as shown **(b)**; represents three independent experiments and shown as average ± S.D. **(c)** HeLa-H4-pEGFP stable cells were transiently transfected with empty vector (1 μg), pSB-miR-133b (1 μg), or pSB-miR-133b (1 μg) and pCMV-Myc-CAN/Nup214 (1 μg) and synchronized. Medium was replaced with phenol red-free medium and visualized under time lapse fluorescence microscope. ‘0.00’ time-point is that time (8 h post-release) when chromosomes for each of the three treated condition were found to be aligned at the metaphase plate. Representative frozen images are shown and time stamps indicated. Scale bar represents 5 μm. Images represent 63× magnification.

Third, we scored the mitotic indices (MI) of HCT116 cells treated with miR-133b based on the p-H3-FITC positive signal. The data supported the above findings in that the MI was higher for cells treated with miR-133b (49%) as compared to vector-treated cells (7.5%) at the 10 h time point (Figure 4a and b). As before, co-expression of Nup214 was able to retrieve the MI to a significant extent (18.6%) (Figure 4a and b). We also supplemented our results with time-lapse monitoring of live Hela-H4-pEGFP cells transfected with miR-133b. The results illustrated that in cells treated with miR-133b, mitosis remained blocked (Figure 4c, middle row and Additional file 6B) even as vector-treated (Figure 4c, top row and Additional file 6A) and Nup214-reconstituted cells (Figure 4c, bottom row and Additional file 6C) proceeded to anaphase and further, within 1 h of monitoring.

The above results fostered the idea that some mitotic factor essential to cell cycle progression may be unable to enter the nucleus in presence of ectopic miR-133b and consequent absence of or low Nup214. To find out whether this possible mitotic cargo of Nup214 functions in the early or late mitotic phase, we followed the levels of cyclinA (whose degradation is an early mitotic marker) in synchronized UPCI:SCC084 as well as HCT116 cells transfected with miR-133b. Indeed, we found that while for vector-transfected UPCI:SCC084 cells cyclinA levels peaked at 4 h post-release (Figure 3c, left panel and f), ectopic miR-133b seemed to bring about the stabilization of the protein till around the 10 h time-point (Figure 3c, middle panel and f). Moreover, similar to the observations with cyclinB1, here too, ectopic expression of Nup214 along with miR-133b was able to reconstitute the cyclic pattern of cyclinA (Figure 3c, right panel and f). Additionally, this pattern of cyclinA degradation, stabilization by miR-133b and rescue by Nup214 was reproduced in HCT116 cells once again (Figure 3g and j). Hence, these results further bolstered our hypothesis that miR-133b arrests cells in early mitosis probably by disturbing the import/export of some crucial mitotic factor through the downregulation of Nup214.

### miR-133b-induced disturbance in mitotic progression causes chromosomal abnormalities

**Figure 5:**
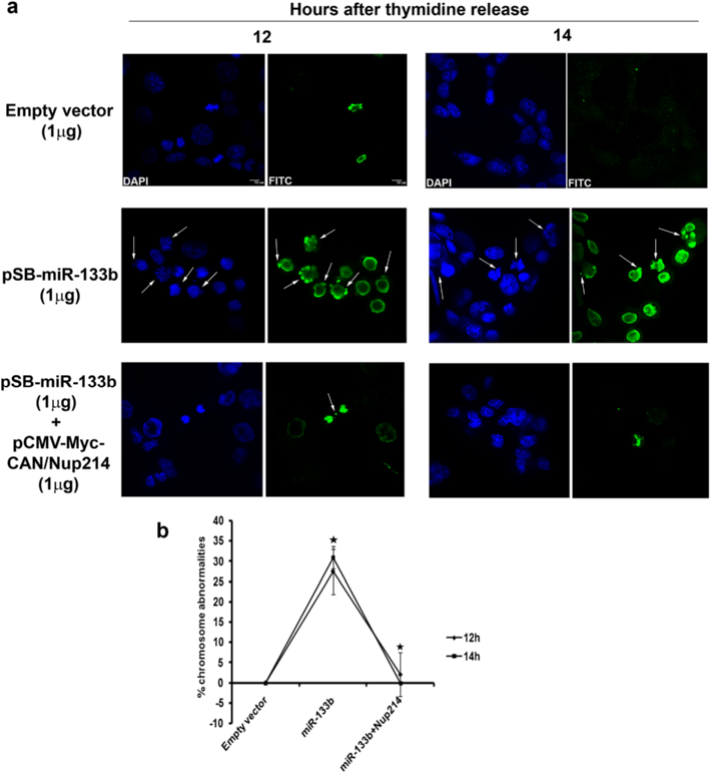
**Excess miR-133b enhances chromosomal abnormalities. (a)** and **(b)** HCT116 cells were transiently transfected with empty vector (1 μg), pSB-miR-133b (1 μg), or pSB-miR-133b (1 μg) and pCMV-Myc-CAN/Nup214 (1 μg) and synchronized. Immuno-staining was done with antibody against p-H3 and nuclei were stained with DAPI at 12 and 14 h from second thymidine release. Cells were visualized under a fluorescence microscope. **(a)** Representative images are shown and the defects indicated by white arrows. Scale bar represents 10 μm. Images represent 60× magnification. **(b)** Percentages of mitotic abnormalities were calculated and plotted as shown. Data represents three independent experiments and shown as average ± S.D.

We next hypothesised that miR-133b might give birth to chromosomal abnormalities as a consequence of the mitotic arrest. Upon addressing this, synchronized HCT116 cells revealed that chromosomal abnormalities were indeed strikingly conspicuous in miR-133b overproducing cells at the 12 h and 14 h time points (from release) as compared to vector-transfected cells (Figure 5a, 1^st^ and 2^nd^ rows and b). P-H3 immuno-staining in these abnormal chromosomes gave further credence that it is the miR-133b-mediated mitotic perturbation that induces chromosomal aberrations. Besides, ectopic expression of Nup214 along with miR-133b was also able to revoke the rise in chromosomal aberrations that was observed in presence of miR-133b alone, thus establishing the specificity of our results (Figure 5a, 3^rd^ row and b). Collectively, these data confirm that miR-133b induces chromosomal abnormalities in a Nup214-specific manner.

### miR-133b-mediated mitotic defects leads to cell death via apoptosis

**Figure 6:**
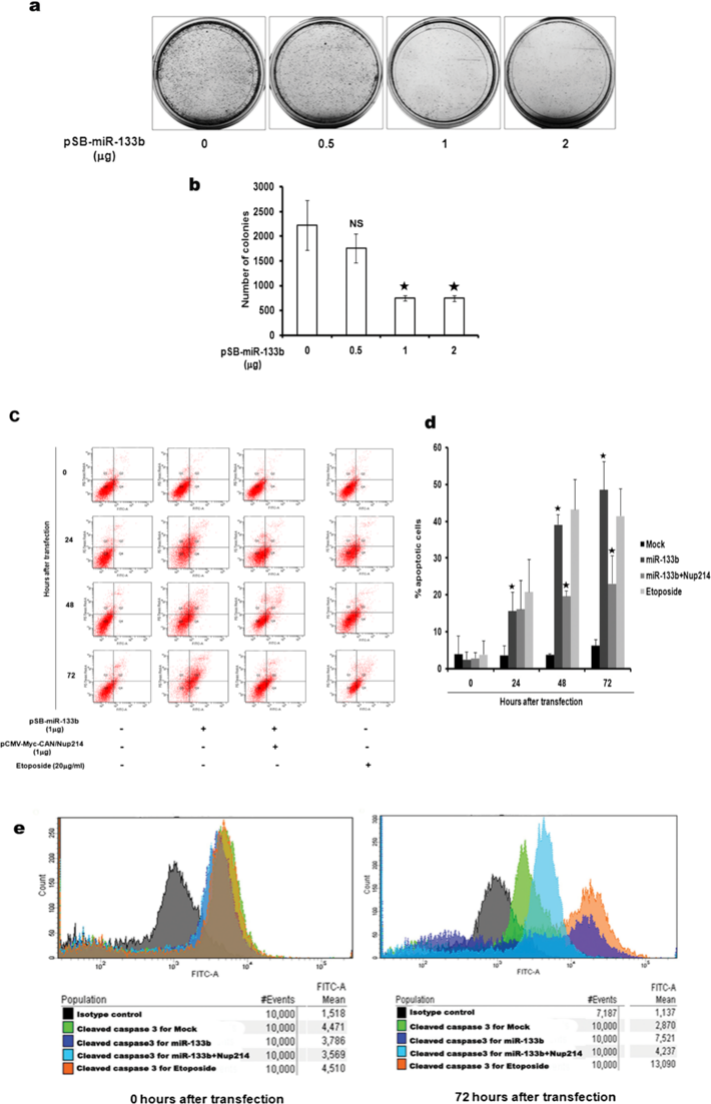
**miR-133b-mediated mitotic delay leads to cell death. (a)** and **(b)** Ectopic miR-133b affects clonogenicity of cells. UPCI:SCC084 cells were seeded at a density of 10^3^ and transiently transfected with 0, 0.25, 0.5, 1 and 2 μg pSB-miR-133b. Colonies were stained with methylene blue after a week. **(a)** Representative images are shown. **(b)** Colonies were counted from **(a)** and plotted as shown. **(c)** and **(d)** Excess miR-133b induces apoptosis. 10^5^ UPCI:SCC084 cells were transiently transfected with empty vector (1 μg), pSB-miR-133b (1 μg), or pSB-miR-133b (1 μg) and pCMV-Myc-CAN/Nup214 (1 μg). At 0, 24, 48 and 72 h post-transfection, cells were subjected to annexin V-FITC/PI staining followed by flow cytometry analysis. **(e)** Ectopic miR-133b elevates cleaved caspase3 levels. 10^5^ UPCI:SCC084 cells were transiently transfected with empty vector (1 μg), pSB-miR-133b (1 μg), or pSB-miR-133b (1 μg) and pCMV-Myc-CAN/Nup214 (1 μg). Cells were fixed, permeabilized and stained with control antibodies or cleaved caspase3 antibody at 0 and 72 h post-transfection followed by flow cytometric analysis. Colour codes of histograms and mean FITC values are indicated. For **(b)**-**(d)**, data represent three independent experiments and shown as average ± S.D. For **(a)** and **(e)**, images are representative of three independent experiments.

Finally, we examined the phenotypic consequence of the miR-133b-mediated Nup214 downregulation and the following mitotic disturbances by studying its effect on the clonogenicity of UPCI:SCC084 cells. According to our results, after a week, the miR-133b overproducing cells gave rise to significantly less number of colonies than those formed by vector-transfected cells (Figure 6a and b). This indicated that miR-133b might reduce cell viability, probably in part, due to the enhancement of chromosomal abnormalities following the mitotic progression defects shown before.

**Figure 7:**
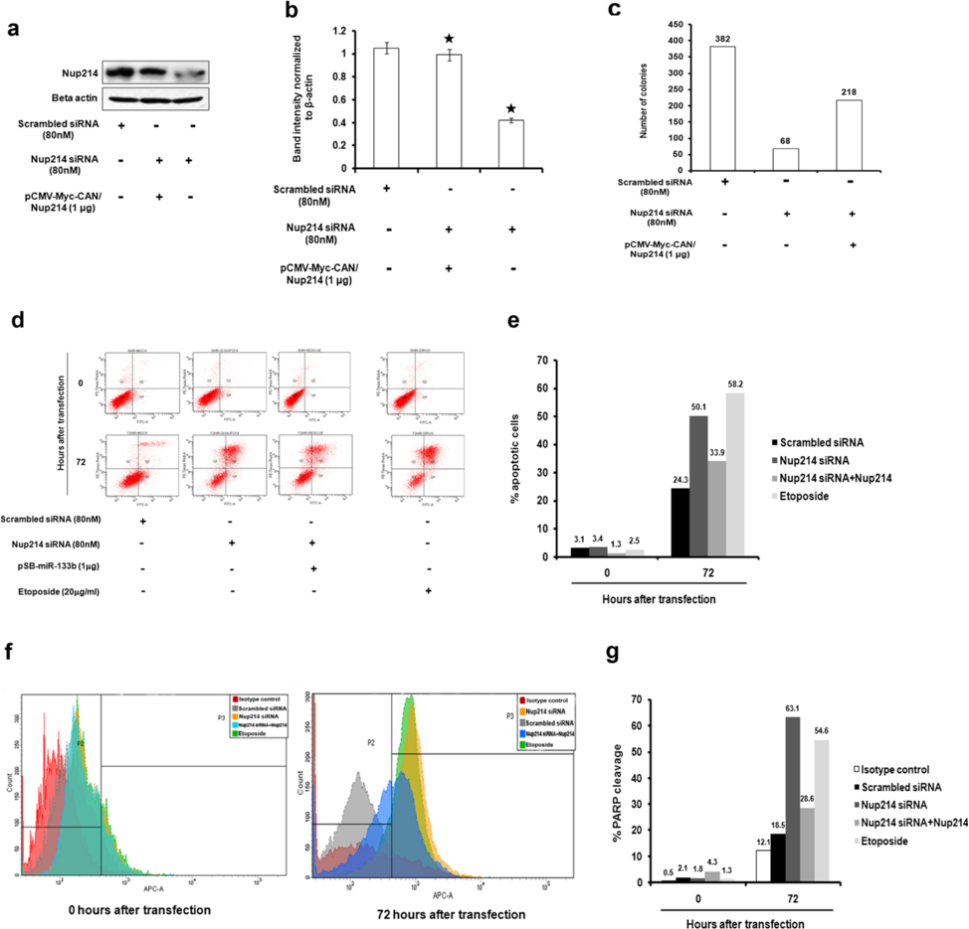
**NUP214 knockdown by siRNAs attests miR-133b-induced phenotypes. (a)** Representative picture showing Nup214 knockdown by specific siRNAs. UPCI:SCC084 cells were transiently transfected with scrambled siRNA (80 nM), Nup214-specific siRNAs (80 nM), or Nup214-specific siRNAs (80 nM) and pCMV-Myc-CAN/Nup214 (1 μg). Cell lysates were prepared followed by Western blot with antibodies against Nup214 and β-actin. **(b)** Densitometric values of bands were normalized to those of β-actin by ImageJ and plotted. For **(a)** and **(b)**, data represent three independent experiments and shown as average ± S.D. **(c)** Nup214 knockdown represses colony forming potential of cells. UPCI:SCC084 cells were seeded at a density of 10^3^ and transiently transfected with scrambled siRNA (80 nM), Nup214-specific siRNAs (80 nM), or Nup214-specific siRNAs (80 nM) and pCMV-Myc-CAN/Nup214 (1 μg). Colonies were stained with methylene blue after a week, counted and plotted as shown. **(d)** and **(e)** Nup214 knockdown induces apoptosis. 10^5^ UPCI:SCC084 cells were transiently transfected with scrambled siRNA (80 nM), Nup214-specific siRNAs (80 nM), or Nup214-specific siRNAs (80 nM) and pCMV-Myc-CAN/Nup214 (1 μg). At 0 and 72 h post-transfection, cells were subjected to annexin V-FITC/PI staining followed by flow cytometry analysis. Representative images are shown **(d)** and data plotted **(e)**. **(f)** and **(g)** Nup214 knockdown elevates cleaved PARP levels. 10^5^ UPCI:SCC084 cells were transiently transfected with scrambled siRNA (80 nM), Nup214-specific siRNAs (80 nM), or Nup214-specific siRNAs (80 nM) and pCMV-Myc-CAN/Nup214 (1 μg). Cells were fixed, permeabilized and stained with control antibodies or cleaved PARP antibody at 0 and 72 h post-transfection followed by flow cytometric analysis. Color codes of histograms are indicated in representative images **(f)**. Data was plotted as shown **(g)**.

For a clearer insight into this observation, however, it was interesting to probe whether the reduction in colony number was due to apoptotic cell death and if so, whether the viability could be retrieved upon ectopic Nup214 expression. Toward this, we performed apoptosis assays in miR-133b-transfected UPCI:SCC084 cells in two different ways. Firstly, time kinetic FACS analysis of annexinV/propidium iodide (PI) stained cells revealed that cells indeed underwent programmed cell death 72 h after miR-133b treatment (39.15%) as compared to vector-treated cells at the same time point (3.9%) (Figure 6c and d). Secondly, evaluation of caspase3 cleavage using flow cytometry was carried out in the above cells. Our results indicate that there is an elevation in caspase3 activation about 72 h post miR-133b transfection in comparison to vector-treated cells at the same time point (Figure 6e). Interestingly, Nup214 co-expression was able to prevent apoptosis by miR-133b alone in both the above experiments (Figure 6c-e). A strong corroboration for these results was achieved when on one hand, specific knockdown of Nup214 in UPCI:SCC084 cells with a pool of siRNAs (Figure 7a and b) gave the same phenotype for cell death as was seen with miR-133b (Figure 7c-f). On the other hand, treatment of non-tumoral HEK293 cells with the same dose of miR-133b demonstrated no significant deviations in either colony number (Additional file 7A and B) or in percentage of apoptotic cells (Additional file 7C and D) when compared to control vector-treated cells. Collectively, these results confirm our view that cells are rendered non-viable by miR-133b in a Nup214-specific manner.

## Discussion

We have validated here that the human miR-133b can specifically repress the nucleoporin proto-oncogene NUP214. miR-133b remains downregulated in head and neck, bladder, colorectal, gastric, lung and prostate cancers while it is upregulated in cervical cancer [26]. This miRNA is also reported to be tumour suppressive in esophageal squamous cell carcinoma [27]. Furthermore, miR-133b has been shown to target anti-apoptotic genes to mediate death-receptor-induced apoptosis [28]. The present study further illustrates that the miR-133b-mediated Nup214 downregulation delays cells in the early mitotic phase and thus leads to aberrant chromosome segregation and cell death. Importantly, from our observations we make an intriguing hypothesis that the import of one or more mitotic proteins (that is/are cell cycle regulated) may be dependent on the levels of Nup214 and hence, on the levels of miR-133b. Our study highlights this possibility through a hitherto unknown miRNA-regulated association of NUP214 with proper mitotic progression.

It was previously reported that NUP214-depleted embryo cells arrested in the G2 phase of the cell cycle [21] while over-expressing cells arrested in G0 [22]. In our attempt to uncover the downstream functional effects of Nup214 downregulation by miR-133b, we also observed a similar G2-M arrest in cells overproducing miR-133b by flow cytometric analysis. The accumulation of cyclinA in miR-133b treated synchronised cells was also able to prove an early mitotic arrest. Furthermore, co-transfection with Nup214 expression-plasmid was able to rescue the degradation of both cyclinA and cyclinB1, dephosphorylation of p-H3 and the MI thus ascertaining the specificity of this interaction. Moreover, the accumulation of cyclinB1 and p-H3 as well as increased MI endorsed the involvement of one or more SAC proteins in this pathway. Notably, because Nup214 lacks its 3′UTR in the expression-vector, co-expression with miR-133b did not alter Nup214’s ability to change the cellular phenotype. This also indicates that other known targets of miR-133b (like CXCR4 [29]) might not play any role in the NUP214-mediated phenotype observed here. To the best of our knowledge, Mad1 is the only SAC protein with known interactions with different Nups across species other than humans [30,31]. Nup88 and hCRM1 also have been previously identified as Nup214 interacting proteins [32]. Interestingly a study of substrate transport through the NPC upon NUP214 depletion revealed that the import of nuclear localization sequence (NLS)-containing proteins into the nucleus was impaired [21]. However, in the absence of concrete evidence for known associations of Nup214 with any of the SAC proteins, for the time being we need to leave it to experimental investigations for further elucidation and explanation of our observations.

To be able to direct the mitotic perturbations to logical explanations, it is incumbent on us to question the sub-cellular localization and distinct functions of Nup214 at mitosis. As mentioned in the Background section, Nup214 exhibits dual citizenship – being relocated from the nuclear pores at interphase to the spindles at mitosis. Not much is known about this, however, in a study by Lussi *et al.* [30], it has been shown that another member of the Nup family, Nup153, can associate with Mad1 (a mitotic protein). However, this role of Nup153 in mitosis was found to be independent of its role in nucleocytoplasmic transport. Because the localization of Nup214 at the spindles during mitosis is as yet unexplained, we suggest a similar situation wherein there may arise two possibilities at the initiation of mitosis: (1) the early mitotic arrest might occur due to defective nuclear transport of some protein/factor (discussed in the Results section) as a result of Nup214 downregulation or (2) Nup214 downregulation may cause mitotic arrest as a result of some altered transport-independent event, for example, defective NEBD, etc. (we mention NEBD because destruction of cyclinA is known to start minutes after NEBD takes place [33]). According to our observations, the former seems to be more plausible, stemming from the fact that cyclinA, cyclinB1 and p-H3 are stabilized upon Nup214 repression by miR-133b. Nevertheless, the latter possibility also demands to be probed by valid experiments.

An earlier report has shown that abolishment of Nup214 from the spindles during mitosis results in chromosome separation defects and aneuploidy, multinucleate structures being the most dramatic [23]. In this context, altered levels of another Nup, Nup153, has also been implicated in the formation of multipolarization and multilobulation of cells [30]. Similarly, our study too, has shown chromosome defects upon Nup214 downregulation by miR-133b- particularly some which appear like “flower cells” with greatly lobulated nuclei (Figure 5a, 2^nd^ row, 12 h time-point) as described by the same authors [30]. miR-133b is reported in death-receptor-induced apoptosis in HeLa cells and *ex vivo* models of pancreatic cancer [28]. We too, observed ectopic miR-133b as well as Nup214 siRNA-mediated induction of apoptotic cell death in UPCI:SCC084 cells. Importantly, incidence of both chromosomal defects as well as apoptosis was greatly reduced upon co-expression of Nup214 along with miR-133b or the siRNA. Hence, we speculate that the late mitotic exit leads to erroneous chromosome segregation and abnormal nuclear structures further leading to cell death, the phenomena being specific to Nup214 in our study.

While the functions of NPCs in transport are well established, coupling of the nuclear transport machinery to processes that control chromosome segregation during mitosis, including the SAC, is still an emerging area of investigation. Our study contributes by involving miRNA-mediated regulation in this process. These findings might also help to understand the molecular mechanisms that contribute towards the high degree of CIN associated with various cancers. In fact, given that miR-133b is downregulated in HNSCC (and many other cancers), our study has significant therapeutic implications- (1) miR-133b mimics could potentially be used to alleviate the oncogenic effects of endogenous Nup214 that lead to malignancies, (2) molecules other than miRNA mimics such as small molecules, nanoparticles or other silencing RNAs could also be used against Nup214. More importantly, the preliminary observation that ectopic miR-133b does not alter the fate of non-tumoral cells also makes it a good therapeutic candidate. Moreover, the fact that our observations have been validated in cell lines other than UPCI:SCC084 (like HCT116 and HeLa) underscores a broader functionality of these mechanisms in cancers. It should be mentioned though that in colorectal cancer, miR-133b has been shown to block cell proliferation by directly targeting the c-MET protein [34]. However, here we have defined Nup214 as a novel direct target of the miRNA and extended our study to more than one cell system.

## Conclusions

In summary, (1) the nucleoporin gene NUP214 is a novel target of miR-133b, (2) Nup214 downregulation brings about early mitotic delay and (3) this delay gives rise to chromosomal segregational defects and eventual cell death. All these cellular effects of miR-133b appear to be Nup214-specific. However, it is crucial to bear in mind that miR-133b has other known and unknown targets across the cell cycle whereas NUP214 regulation could also be controlled by hitherto un-validated miRNAs. Hence, a systemic study of miRNAs involved in the regulation of Nup214 and its interacting partners is warranted to fully understand the impact of miRNAs on the development and progression of cancer. An in-depth study of Nup214 localization and its precise functions at its individual sites is also crucial to further our understanding of this phenomenon.

## Methods

### Cell culture, synchronization, drug treatment and transfection

HCT116, SCC25, SW480 and HEK293 cell lines were purchased from American Type Culture Collection (Manassas, VA, USA) and HepG2 cell line was gifted by Dr. Samit Adhya (Indian Institute of Chemical Biology, India). Human oral cancer cell line UPCI:SCC084 was a kind gift from Dr. Susanne M. Gollin (University of Pittsburgh, USA). HeLa-H4-pEGFP stable cell line was developed as reported previously [35]. All cell lines were cultured in Dulbecco’s Modified Eagle’s Medium (DMEM; Invitrogen, Carlsbad, CA, USA) supplemented with 10% fetal calf serum and antibiotics (1% Pen Strep Glutamine and 0.006% Gentamicin, Invitrogen) at 37°C in a 5% CO_2_ incubator. For synchronization, cells were treated with thymidine (2.5 mM; USB, Cleveland, USA) for 16 h followed by an 8 h release. A second thymidine treatment was given for another 22 h. Cells were released in thymidine free complete medium and harvested at various time-points following this double thymidine block. Transient transfections were done with various plasmids, miRNA inhibitors and siRNAs in different cell lines using Lipofectamine 2000 reagent (Invitrogen) according to the manufacturer’s protocol. All transient transfections were performed for 48 h, except in case of synchronization experiments. Transfection of synchronized cells was done 6 h before first thymidine addition. For induction of apoptosis by etoposide (Sigma) and camptothecin (Sigma), cells were treated with the drug (20 μg/ml for 72 h and 3 μM for 24 h, respectively) for the time-periods mentioned.

### Plasmids, miRNA inhibitors and siRNAs

The cloning of pSB-miR-133b (human precursor miR-133b; chromosome 6: 52011721–52015839, + strand) is described by Bhattacharjya *et al.* [20]. The NUP214 3′ UTR (NM_005085.2, +6355 to +6475) was amplified from human genomic DNA with the primers listed in Additional file 8. This region was cloned into the linearized pMIR-REPORT vector (Applied Biosystems, Foster City, USA) downstream of the luciferase gene using the MluI and HindIII restriction sites (New England Biolabs, Beverly, MA). It is referred to as pSB-NUP214/3′UTRLuc in the text. The mutant version of this clone was constructed using the primers listed in Additional file 8 and Quikchange XL site-directed mutagenesis kit (Stratagene, La Jolla, CA, USA) according to the manufacturer’s protocol. It is referred to as pSB-NUP214/3′UTRMutLuc in the text. Full length Myc-tagged Nup214 expression-plasmid pCMV-Myc-CAN/Nup214 was a kind gift from Dr. Toshimi Michigami (Osaka Medical Center for Maternal and Child Health, Japan). Anti miR-133b (Ambion, Austin, TX, USA) was used at a final concentration of 20 nM. siRNAs directed against Nup214 (Santa Cruz Biotechnology) as well as scrambled control (Ambion) were used at a final concentration of 80 nM.

### Quantitative Real-Time PCR

Total RNA from different cell lines, oral swab and tissue specimens was isolated using TRIZOL (Invitrogen) according to the manufacturer’s protocol. 5 μg isolated RNA was treated with DNase (Promega, Madison, USA) and 1 μg of this DNase treated RNA was used for cDNA preparation using random hexamer (Invitrogen) and MMLV-RT (Promega). For miRNAs, 200 ng isolated RNA was used for cDNA preparation in a master mix containing stem-loop primers specific for the desired miRNAs (Sigma) as given in Additional file 9, dNTPs (Invitrogen) and MMLV-RT (Promega). Real time PCR was performed in the 7500 Fast Real-Time PCR System (Applied Biosystems) using power SYBR Green PCR Master Mix (Applied Biosystems). The comparative threshold cycle method (ΔΔCt) was used to quantify relative amounts of product transcripts with GAPDH or 18S rRNA (for mRNAs) and hsa-miR-17-5p or U6 snRNA (for miRNAs) as endogenous reference controls. Primer sets are listed in Additional file 9. Fold activation values were calculated as mean of three independent experiments.

### Western blotting and antibodies

Whole cell lysates having equal protein concentrations were resolved by SDS/PAGE (6%–12% gel) and transferred onto a PVDF membrane (Millipore, Billerica, USA). Various primary antibodies used are goat polyclonal NUP214, rabbit polyclonal p-H3, rabbit polyclonal cyclinA, goat monoclonal Bub1, rabbit polyclonal Nup98 (Santa Cruz Biotechnology, CA, USA), mouse monoclonal cyclinB1 (Cell Signaling Technology, Beverly, MA, USA) and mouse monoclonal β-actin antibody (Sigma). Bands were detected using Super Signal West Pico chemiluminescent substrate (Thermo Scientific, Rockford, IL, USA) after treating with HRP-conjugated secondary antibody (Sigma).

### Luciferase assay

Cells were lysed with luciferase cell culture lysis reagent supplied with the luciferase assay kit (Promega). Following a short vortex, whole cell lysates were centrifuged at 4°C at 13,000 rpm for 2 min and 15–30 μl of supernatants was mixed with 30–60 μl of luciferase assay substrate. Luminescence was measured as relative luciferase unit (RLU) in GLOMAX luminometer (Promega). Total protein concentration in each lysate was measured by Bio-Rad protein assay reagent (Bio-Rad Laboratories, CA, USA) and then used to normalize the luciferase activity of each lysate. Fold activation values were calculated as mean of three independent experiments.

### Cell cycle and apoptotic assays

UPCI:SCC084 cells were synchronized and after release, approximately 10^6^ cells were harvested at respective time-points and resuspended in 0.25 ml of cold phosphate buffered saline (PBS). Cells were fixed by adding cold 70% ethanol dropwise into the samples while vortexing gently and then incubated at 4°C for minimum 24 h. After fixation, cells were resuspended in 1 ml PBS containing 100 μg/ml PI (Sigma) and 20 μg/ml RNase A (Invitrogen). Fixed cells were kept at room temperature for 40 min and then analysed by FACS (BD FACSARIA™ III, Becton-Dickinson, San Jose, CA, USA).

For annexin V-PI binding assay, UPCI:SCC084 or HEK293 cells were seeded at a density of 10^5^ cells and transfected as mentioned. Apoptosis was measured at the indicated time-points using FITC AnnexinV Apoptosis Detection Kit I (BD Pharmingen) according to the manufacturer’s protocol. Analysis was done by LSRFortesa (Becton-Dickinson).

To measure caspase3/PARP cleavage, 10^5^ UPCI:SCC084 cells were seeded and transiently transfected as mentioned. At indicated time-points, cells were fixed with 4% paraformaldehyde, permeabilized by fluorescence-activated cell sorter permeabilizing solution (BD Biosciences, San Jose, CA, USA). Before staining, permeabilized cells were treated with heat-inactivated 2% normal goat serum to block non-specific staining. Cells were then stained with normal rabbit sera or rabbit anti-human cleaved caspase3/PARP antibody (Cell Signaling Technology). After washing, cells were incubated with multiple adsorbed FITC-conjugated secondary antibody (goat anti-rabbit immunoglobulin; BD Biosciences), washed and analysed by LSRFortesa (Becton-Dickinson).

For colony formation assay, 10^3^ UPCI:SCC084 or HEK293 cells were seeded and transiently transfected with indicated concentrations of plasmid. After 7 days, cells were stained with 0.2% Methylene Blue [Sisco Research laboratories (SRL), Mumbai, India] and washed with distilled water. The colonies were then counted and the number of colonies was calculated as mean of three independent experiments.

### Immuno-fluorescence

Synchronized HCT116 cells were harvested at different time-points (as mentioned) after thymidine release. They were then fixed with ice-cold acetomethanol (1:1) and permeabilized with 0.03% saponin (Calbiochem, Darmstadt, Germany). After blocking in 3% BSA, rabbit polyclonal antibody against p-H3 (Santa Cruz Biotechnology) was added. Secondary antibody conjugated with FITC (Sigma) was added and the nuclei were stained with (DAPI) (Invitrogen). Slides were observed under a fluorescence microscope (Leica DM 3000, IL, USA). Abnormal chromosomes were counted among 100–200 cells each time from three independent experiments and plotted as percent abnormality.

### Live cell microscopy

HeLa-H4-pEGFP cells were grown in 35 mm dishes with glass bottoms, transiently transfected (as mentioned) and synchronized. DMEM medium was replaced with phenol red-free DMEM medium (Invitrogen). Cells were monitored for duration in mitosis starting from 8 h post thymidine release under an inverted fluorescent microscope with live cell chamber (Leica DMI6000) at 37°C and 5% CO_2_. Images with a z step size of 500 nm were captured after every 10 minutes with both DIC and green channels using a 63× oil immersion objective.

### Tumour samples

Matched oral tumour (n = 10) and normal (n = 10) tissues were obtained from the hospital section, Chittaranjan National Cancer Institute (CNCI), Kolkata, India. Prior to sample collection, written informed consent was taken from each individual and approved by the Research Ethics Committee of CNCI. The pathological history of the tumours is provided in Additional file 4.

### Bioinformatic and statistical analysis

Target prediction of miR-133b was done by miRBase (version 17). Complementarity between NUP214-3′UTR and the seed sequence of miR-133b was obtained from RNAhybrid (version 2.1) [36] (http://bibiserv.techfak.uni-bielefeld.de/rnahybrid/). Oncomine 4.4 research edition database [37] (http://www.oncomine.org/resource/login.html) was used for the dataset of Nup214 over-expression in primary tumours. Cancer versus normal datasets of Nup214 over-expression with fold change ≥1.5 and p-value ≤0.05 were selected. Densitometric scanning for Western blots was done by ImageJ software (http://rsb.info.nih.gov/ij/index.html). Students *t* test was used to determine statistically significant differences which were defined by 2-sided p < 0.05. GraphPad Prism 5 was used to perform Mann Whitney *t*-test in order to determine significant differences in miR-133b and NUP214 expressions between individual groups (normal and tumour).

## Additional files

Additional file 1: ***NUP214*** **is a putative target of miR-133b.** (A) The nucleotide position 9-36 of *NUP214* 3′ UTR is the recognition site for miR-133b as predicted by RNAhybrid. Letters in upper case denote the seed region. (B) The predicted stable RNA-RNA duplex formed by the binding of human miR-133b to the 3′UTR of *NUP214* as given by RNAhybrid. The RNA strand in green represents miR-133b and the RNA strand in red represents position 9-36 of *NUP214* 3′UTR. mfe – minimum free energy. (C) Nup214 expression is upregulated in different cancers. Expression data of Nup214 in 23 different cancers from Oncomine database were analyzed. Cancer versus normal datasets of Nup214 over-expression with fold change ≥1.5 and p-value ≤0.05 were selected. Bars represent fold over-expression of Nup214 in these cancers. Electronic supplementary material.

Additional file 2: **Analysis of Oncomine dataset shows that Nup214 expression is upregulated in 23 different cancers in comparison to normal tissues (refer to Additional file** 1 **C).** Cancer versus normal datasets of Nup214 over-expression with fold change ≥1.5 and p-value ≤0.05 were selected.

Additional file 3: **Expression values for individual patient samples.**

Additional file 4: **Pathological details of tumours.**

Additional file 5: **miR-133b targets** ***NUP214*** **specifically.** (A) Dose-dependent increase in miR-133b expression upon transient transfection of UPCI:SCC084 cells with pSB-miR-133b (refer to Figure 1d). (B) ImageJ analysis of Nup214 bands in Figure 1e normalized to β-actin. (C) Representative picture showing Nup98 protein level unaffected upon ectopic miR-133b expression. UPCI:SCC084 cells were transiently transfected with 0 and 1 μg pSB-miR-133b. Lysates were prepared followed by Western blot with antibodies against Nup98 and β-actin. (D) BUB1 transcript level is unaffected by ectopic miR-133b. UPCI:SCC084 cells were transiently transfected with 0, 0.25, 0.5, 1 and 2 μg pSB-miR- 133b. Total RNA isolated was reverse transcribed and cDNA subjected to RT-PCR using BUB1-specific primers. (E) Representative picture showing Bub1 protein level unaffected upon ectopic miR-133b expression. UPCI:SCC084 cells were transiently transfected with 0, 0.25, 0.5, 1 and 2 μg pSB-miR-133b. Lysates were prepared followed by Western blot with antibodies against Bub1 and β-actin. (F) ImageJ analysis of Bub1 bands in (E) normalized to β-actin. (G) NUP214 transcript level remains unaltered in presence of unrelated miR-125b. UPCI:SCC084 cells were transiently transfected with 0, 0.25, 0.5 and 1 μg miR-125b expression-plasmid. Total RNA isolated was reverse transcribed and cDNA subjected to RT-PCR using NUP214-specific primers. (H) Representative picture showing Nup214 protein level unaffected upon ectopic expression of miR-125b. UPCI:SCC084 cells were transiently transfected with 0, 0.25, 0.5 and 1 μg miR-125b expression-plasmid. Lysates were prepared followed by Western blot with antibodies against Nup214 and β-actin. (I) ImageJ analysis of Nup214 bands in (H) normalized to β-actin. For (D) and (G), relative expression values were normalized to those of GAPDH. For (A), (B), (D), (F), (G) and (I), data represent three independent experiments and are shown as average ± S.D. For (C), (E), (F) and (H), images are representative of three independent experiments.

Additional file 6: **miR-133b-mediated Nup214 repression lengthens mitotic duration.** (A), (B) and (C) HeLa-H4-pEGFP stable cells were transiently transfected with empty vector (1 μg), pSB-miR-133b (1 μg), or pSB-miR-133b (1 μg) and pCMV-Myc- CAN/Nup214 (1 μg) and synchronized. Medium was replaced with phenol red-free medium and visualized under time lapse fluorescence microscope.

Additional file 7: **Excess miR-133b does not modulate cell fate of non-tumoral cells.** (A) and (B) Ectopic miR-133b does not influence clonogenicity of non-tumoral cells. HEK293 cells were seeded at a density of 103 and transiently transfected with 0 and 1 μg of pSB-miR-133b. Colonies were stained with methylene blue after a week. Representative images are shown (A); colonies were counted from (A) and plotted as shown (B). (C) and (D) Excess miR-133b does not induce apoptosis in non-tumoral cells. 105 HEK293 cells were transiently transfected with 0 and 1 μg of pSB-miR-133b. At 72 h post-transfection, cells were subjected to annexin V-FITC/PI staining followed by flow cytometry analysis and plotted. For (A) and (C), images are representative of three independent experiments; for (B), data is shown as average ± S.D.

Additional file 8: **Primers used for cloning.**

Additional file 9: **Primers used for RT-PCR.**

## Abbreviations

Nup: Nucleoporin
NPC: Nuclear pore complex
miR: MicroRNA
CIN: Chromosomal instability
SAC: Spindle assembly checkpoint
UTR: Untranslated region
NEBD: Nuclear Envelope breakdown
p-H3: Phosphorylated histone H3
HNSCC: Head and neck squamous cell carcinoma
MI: Mitotic index
