# Supplementary figures and images for: Inhibition of nucleoporin member Nup214 expression by miR-133b perturbs mitotic timing and leads to cell death
Source: Mol Cancer. 2015 Feb 15;14:42. doi: 10.1186/s12943-015-0299-z (PMC4335456; doi:10.1186/s12943-015-0299-z)

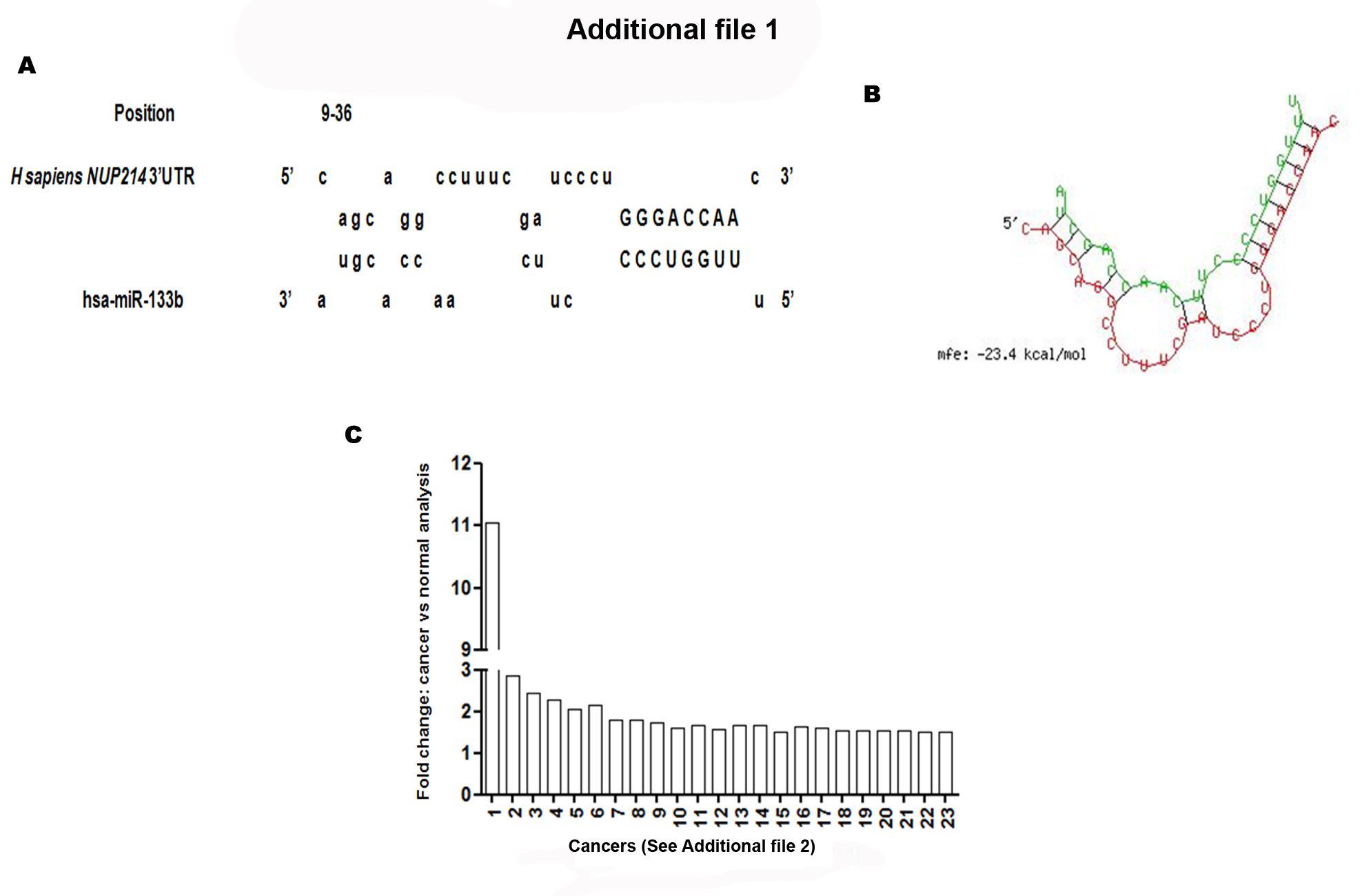

Supplement: Supplementary file 1 — NUP214 is a putative target of miR-133b. (A) The nucleotide position 9-36 of NUP214 3′ UTR is the recognition site for miR-133b as predicted by RNAhybrid. Letters in upper case denote the seed region. (B) The predicted stable RNA-RNA duplex formed by the binding of human miR-133b to the 3′UTR of NUP214 as given by RNAhybrid. The RNA strand in green represents miR-133b and the RNA strand in red represents position 9-36 of NUP214 3′UTR. mfe – minimum free energy. (C) Nup214 expression is upregulated in different cancers. Expression data of Nup214 in 23 different cancers from Oncomine database were analyzed. Cancer versus normal datasets of Nup214 over-expression with fold change ≥1.5 and p-value ≤0.05 were selected. Bars represent fold over-expression of Nup214 in these cancers. Electronic supplementary material. [file 12943_2015_299_MOESM1_ESM.doc]

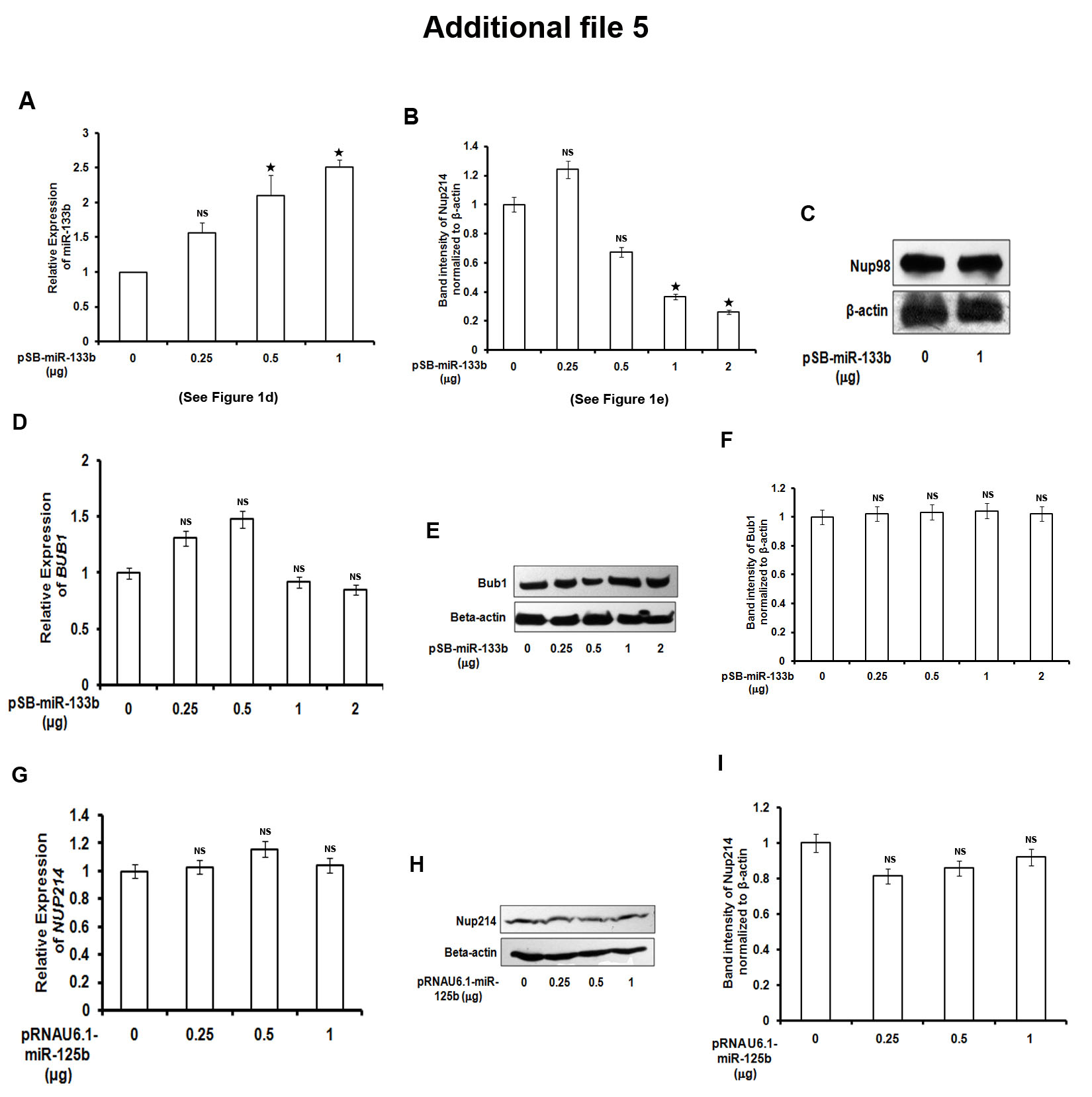

Supplement: Supplementary file 5 — miR-133b targets NUP214 specifically. (A) Dose-dependent increase in miR-133b expression upon transient transfection of UPCI:SCC084 cells with pSB-miR-133b (refer to Figure 1d). (B) ImageJ analysis of Nup214 bands in Figure 1e normalized to β-actin. (C) Representative picture showing Nup98 protein level unaffected upon ectopic miR-133b expression. UPCI:SCC084 cells were transiently transfected with 0 and 1 μg pSB-miR-133b. Lysates were prepared followed by Western blot with antibodies against Nup98 and β-actin. (D) BUB1 transcript level is unaffected by ectopic miR-133b. UPCI:SCC084 cells were transiently transfected with 0, 0.25, 0.5, 1 and 2 μg pSB-miR- 133b. Total RNA isolated was reverse transcribed and cDNA subjected to RT-PCR using BUB1-specific primers. (E) Representative picture showing Bub1 protein level unaffected upon ectopic miR-133b expression. UPCI:SCC084 cells were transiently transfected with 0, 0.25, 0.5, 1 and 2 μg pSB-miR-133b. Lysates were prepared followed by Western blot with antibodies against Bub1 and β-actin. (F) ImageJ analysis of Bub1 bands in (E) normalized to β-actin. (G) NUP214 transcript level remains unaltered in presence of unrelated miR-125b. UPCI:SCC084 cells were transiently transfected with 0, 0.25, 0.5 and 1 μg miR-125b expression-plasmid. Total RNA isolated was reverse transcribed and cDNA subjected to RT-PCR using NUP214-specific primers. (H) Representative picture showing Nup214 protein level unaffected upon ectopic expression of miR-125b. UPCI:SCC084 cells were transiently transfected with 0, 0.25, 0.5 and 1 μg miR-125b expression-plasmid. Lysates were prepared followed by Western blot with antibodies against Nup214 and β-actin. (I) ImageJ analysis of Nup214 bands in (H) normalized to β-actin. For (D) and (G), relative expression values were normalized to those of GAPDH. For (A), (B), (D), (F), (G) and (I), data represent three independent experiments and are shown as average ± S.D. For (C), (E), (F) and (H), images are repr [file 12943_2015_299_MOESM5_ESM.doc]

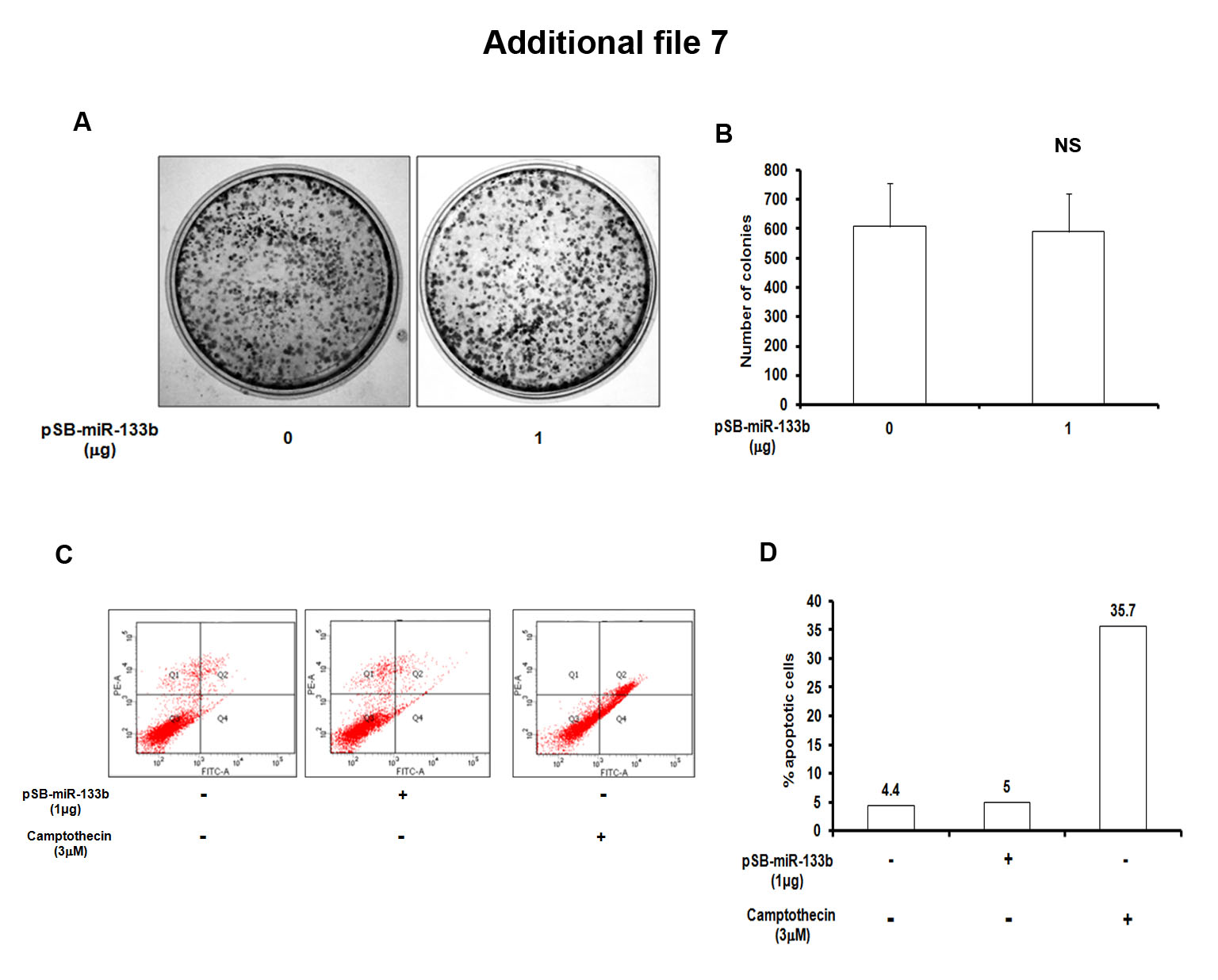

Supplement: Supplementary file 7 — Excess miR-133b does not modulate cell fate of non-tumoral cells. (A) and (B) Ectopic miR-133b does not influence clonogenicity of non-tumoral cells. HEK293 cells were seeded at a density of 103 and transiently transfected with 0 and 1 μg of pSB-miR-133b. Colonies were stained with methylene blue after a week. Representative images are shown (A); colonies were counted from (A) and plotted as shown (B). (C) and (D) Excess miR-133b does not induce apoptosis in non-tumoral cells. 105 HEK293 cells were transiently transfected with 0 and 1 μg of pSB-miR-133b. At 72 h post-transfection, cells were subjected to annexin V-FITC/PI staining followed by flow cytometry analysis and plotted. For (A) and (C), images are representative of three independent experiments; for (B), data is shown as average ± S.D. [file 12943_2015_299_MOESM7_ESM.doc]
