# Supplementary material for: Inhibition of nucleoporin member Nup214 expression by miR-133b perturbs mitotic timing and leads to cell death
Source: Mol Cancer. 2015 Feb 15;14:42. doi: 10.1186/s12943-015-0299-z (PMC4335456; doi:10.1186/s12943-015-0299-z)
Supplement: Supplementary file 2 — Analysis of Oncomine dataset shows that Nup214 expression is upregulated in 23 different cancers in comparison to normal tissues (refer to Additional file 1 C). Cancer versus normal datasets of Nup214 over-expression with fold change ≥1.5 and p-value ≤0.05 were selected. [file 12943_2015_299_MOESM2_ESM.doc]

| **Additional File 2. List of cancers in which Nup214 is upregulated** (see Additional file 1C) | | | | |
| --- | --- | --- | --- | --- |
| **Serial No.** | **Cancer group** | **Cancer type** | **Fold change** | **P value** |
| 1 | FriersonHF salivary gland | Salivary gland adenoid cystic carcinoma | 11.053 | 4.54E-04 |
|  |  |  |  |  |
| 2 | Derrico gastric | Gastric inrestinal type adenocarcinoma | 2.863 | 4.56E-08 |
| 3 |  | Gastric mixed adenocarcinoma | 2.443 | 0.002 |
|  |  |  |  |  |
| 4 | Pomeroy brain | Malignant glioma, NOS | 2.286 | 0.012 |
| 5 |  | Atypical teratoid/rhabdoid tumour | 2.069 | 0.02 |
|  |  |  |  |  |
| 6 | Wang gastric | Gastric cancer | 2.157 | 0.01 |
|  |  |  |  |  |
| 7 | Santegoets vulva | Vulvar intraepithelial neoplasia | 1.803 | 4.19E-04 |
|  |  |  |  |  |
| 8 | Sorlie breast 2 | Fibroadenoma | 1.791 | 0.045 |
|  |  |  |  |  |
| 9 | Yusenko renal | Chromophobe renal cell carcinoma | 1.722 | 0.016 |
| 10 |  | Renal oncocytoma | 1.621 | 0.037 |
|  |  |  |  |  |
| 11 | TCGA brain | Glioblastoma | 1.69 | 0.022 |
| 12 |  | Brain glioblastoma | 1.656 | 1.72E-04 |
|  |  |  |  |  |
| 13 | French brain | Anaplastic oligoastrocytoma | 1.677 | 0.002 |
| 14 |  | Anaplastic oligodendroglioma | 1.568 | 1.44E-04 |
|  |  |  |  |  |
| 15 | Murat brain | Glioblastoma | 1.669 | 7.21E-04 |
|  |  |  |  |  |
| 16 | Liang brain | Oligoastrocytoma | 1.628 | 2.00E-02 |
|  |  |  |  |  |
| 17 | Wurmbach liver | Hepatocellular carcinoma | 1.59 | 2.50E-04 |
|  |  |  |  |  |
| 18 | Riker melanoma | Skin basal cell carcinoma | 1.544 | 0.014 |
|  |  |  |  |  |
| 19 | TCGA breast | Mucinous breast carcinoma | 1.541 | 0.007 |
|  |  |  |  |  |
| 20 | Bredel brain 2 | Anaplastic oligoastrocytoma | 1.531 | 0.047 |
|  |  |  |  |  |
| 21 | Sun brain | Glioblastoma | 1.53 | 2.31E-09 |
|  |  |  |  |  |
| 22 | Sabates-Bellver colon | Rectal adenoma | 1.511 | 0.005 |
|  |  |  |  |  |
| 23 | Varambally prostate | Prostate carcinoma | 1.509 | 0.014 |

**Oncomine thresholds:** p-value≤0.05; fold change=1.5

**Oncomine filters:** cancer vs normal
