# Supplementary material for: Inhibition of nucleoporin member Nup214 expression by miR-133b perturbs mitotic timing and leads to cell death
Source: Mol Cancer. 2015 Feb 15;14:42. doi: 10.1186/s12943-015-0299-z (PMC4335456; doi:10.1186/s12943-015-0299-z)
Supplement: Supplementary file 3 — Expression values for individual patient samples. [file 12943_2015_299_MOESM3_ESM.doc]

| **Additional file 3. Expression values for individual patient samples** | | | | | | |
| --- | --- | --- | --- | --- | --- | --- |
| **Samples** | **ΔCt values for**  **miR-133b in normal**  **(N)** | **ΔCt values for**  **miR-133b in tumour**  **(T)** | **Relative expression**  **(T vs N)** | **ΔCt values for**  **NUP214 in normal**  **(N)** | **ΔCt values for**  **NUP214 in tumour**  **(T)** | **Relative expression**  **(T vs N)** |
| 1 | -5.07 | -2.13 | 0.13 | 6.395 | 7.48 | 0.47 |
| 2 | -8.335 | 4.12 | 0.0002 | 6.685 | 8.68 | 0.25 |
| 3 | -3.49 | -1.335 | 0.22 | 10.15 | 3.855 | 78.5 |
| 4 | -6.245 | 8.625 | 0.00003 | 8.525 | 5.565 | 7.78 |
| 5 | -8.19 | 0.095 | 0.003 | 8.475 | 6.335 | 4.41 |
| 6 | -8.865 | -6.1 | 0.15 | 8.98 | 2.495 | 89.57 |
| 7 | -13.365 | -1.865 | 0.0003 | 8.39 | 3.445 | 30.8 |
| 8 | -9.25 | -1.635 | 0.005 | 9.09 | 5.705 | 10.45 |
| 9 | -11.93 | 2.665 | 0.00004 | 8.31 | 9.555 | 0.42 |
| 10 | -14.205 | -5.83 | 0.003 | 5.44 | 2.63 | 7.01 |
