# Supplementary material for: Inhibition of nucleoporin member Nup214 expression by miR-133b perturbs mitotic timing and leads to cell death
Source: Mol Cancer. 2015 Feb 15;14:42. doi: 10.1186/s12943-015-0299-z (PMC4335456; doi:10.1186/s12943-015-0299-z)
Supplement: Supplementary file 4 — Pathological details of tumours. [file 12943_2015_299_MOESM4_ESM.doc]

| **Additional file 4. Pathological details of tumours** | | | | | |
| --- | --- | --- | --- | --- | --- |
| **Patient ID** | **Age in years/**  **Sex** | **Site** | **Histology** | **Clinical**  **stage** | **Lymph node** |
| 5225 | 45/F | ALV | WDSCC | IV | + |
| 6457 | 45/M | ALV/BM | WDSCC | III | + |
| 4864 | 45/F | BM | WDSCC | IV | + |
| 4155 | 48/M | TNG | MDSCC | II | + |
| 4216 | 40/F | ALV/BM | WDSCC | IV | + |
| 649 | 70/M | RMT | WDSCC | IV | + |
| 4589 | 35/M | ALV/BM | WDSCC | IV | + |
| 4033 | 53/M | BM | MDSCC | IV | - |
| 5721 | 47/M | TNG | PDSCC | III | + |
| 5062 | 52/M | RMT | MDSCC | II | - |

BM, buccal mucosa; ALV, alveolus; TNG, tongue; RMT, retro-molar trigone; WDSCC, well differentiated squamous cell carcinoma; MDSCC, moderately differentiated squamous cell carcinoma; PDSCC, poorly differentiated squamous cell carcinoma
