# Supplementary material for: Inhibition of nucleoporin member Nup214 expression by miR-133b perturbs mitotic timing and leads to cell death
Source: Mol Cancer. 2015 Feb 15;14:42. doi: 10.1186/s12943-015-0299-z (PMC4335456; doi:10.1186/s12943-015-0299-z)
Supplement: Supplementary file 8 — Primers used for cloning. [file 12943_2015_299_MOESM8_ESM.doc]

| **Additional file 8. Primers used for cloning** | | |
| --- | --- | --- |
| Targets | Primers | Annealing temperature |
| *NUP214* 3’ UTR | (F) CGA CGC GTG TCT GTC CAG GGT TTT GGT G  (R) CCC AAG CTT TGT GTT TTG ATG GCA ACG TC | 64^0^C |
| Mutant  *NUP214* 3’ UTR | (F) CAG CAG GCC TTT CGA TCC CTT CGC GCC GCC GCA TCC TCA GCT TC  (R) GAA GCT GAG GAT GCG GCG GCG CGA AGG GAT CGA AAG GCC TGC TG | 60^0^C |
