# Supplementary material for: Inhibition of nucleoporin member Nup214 expression by miR-133b perturbs mitotic timing and leads to cell death
Source: Mol Cancer. 2015 Feb 15;14:42. doi: 10.1186/s12943-015-0299-z (PMC4335456; doi:10.1186/s12943-015-0299-z)
Supplement: Supplementary file 9 — Primers used for RT-PCR. [file 12943_2015_299_MOESM9_ESM.doc]

| **Additional file 9. Primers used for RT-PCR** | | |
| --- | --- | --- |
| Targets | Primers | Annealing temperature |
| *NUP214* | (F) ACC ACT GGA TCC CAA GAG TG  (R) CGA TTG TTG GCT AGG GTG TT | 57^0^C |
| *BUB1* | (F) CAT GCA CAC CCC GCA AAA TGT C  (R) GCA TCT TTG CTG GCC ACT GC | 55^0^C |
| *GAPDH* | (F) ATG GGG AAG GTG AAG GTC CG  (R) GGG TGC TAA GCA GTT GGT | 55^0^C |
| 18S rRNA | (F) TGA CTC TAG ATA ACC TCG GG    (R) GAC TCA TTC CAA TTA CAG GG | 60^0^C |
| U6 snRNA | (F) CTCGCTTCGGCAGCACATATACT  (R) ACGCTTCACGAATTTGCGTGTC | 60^0^C |
| miR-133b | ACA CTC CAG CTG GGT TTG GTC CCC TTC AA | 60^0^C |
| miR-17-5p | ACA CTC CAG CTG GGC AAA GTG CTT ACA GT | 60^0^C |
| Universal reverse primer | TGG TGT CGT GGA GTC G | 60^0^C |
| **Loop primers for miRNA cDNA synthesis** | | |
| miR-133b | CTC AAC TGG TGT CGT GGA GTC GGC AAT TCA GTT GAG TAG CTG GT | 16^0^C |
| miR-17-5p | CTC AAC TGG TGT CGT GGA GTC GGC AAT TCA GTT GAG ACT ACC TG | 16^0^C |
| U6 snRNA | AAA ATA TGG AAC GCT TCA CGA ATT TG | 16^0^C |
